# Applications of organoid technology to brain tumors

**DOI:** 10.1111/cns.14272

**Published:** 2023-05-29

**Authors:** Jie Wen, Fangkun Liu, Quan Cheng, Nathaniel Weygant, Xisong Liang, Fan Fan, Chuntao Li, Liyang Zhang, Zhixiong Liu

**Affiliations:** ^1^ Department of Neurosurgery Xiangya Hospital, Central South University Changsha Hunan China; ^2^ Hypothalamic‐pituitary Research Center Xiangya Hospital, Central South University Changsha Hunan China; ^3^ National Clinical Research Center for Geriatric Disorders Xiangya Hospital, Central South University Changsha Hunan China; ^4^ Academy of Integrative Medicine Fujian University of Traditional Chinese Medicine Fuzhou Fujian China; ^5^ Fujian Key Laboratory of Integrative Medicine in Geriatrics Fujian University of Traditional Chinese Medicine Fuzhou Fujian China

**Keywords:** brain metastases, brain tumor, glioblastoma, medulloblastoma, meningioma, organoid, precision medicine, tumor microenvironment

## Abstract

Lacking appropriate model impedes basic and preclinical researches of brain tumors. Organoids technology applying on brain tumors enables great recapitulation of the original tumors. Here, we compared brain tumor organoids (BTOs) with common models including cell lines, tumor spheroids, and patient‐derived xenografts. Different BTOs can be customized to research objectives and particular brain tumor features. We systematically introduce the establishments and strengths of four different BTOs. BTOs derived from patient somatic cells are suitable for mimicking brain tumors caused by germline mutations and abnormal neurodevelopment, such as the tuberous sclerosis complex. BTOs derived from human pluripotent stem cells with genetic manipulations endow for identifying and understanding the roles of oncogenes and processes of oncogenesis. Brain tumoroids are the most clinically applicable BTOs, which could be generated within clinically relevant timescale and applied for drug screening, immunotherapy testing, biobanking, and investigating brain tumor mechanisms, such as cancer stem cells and therapy resistance. Brain organoids co‐cultured with brain tumors (BO‐BTs) own the greatest recapitulation of brain tumors. Tumor invasion and interactions between tumor cells and brain components could be greatly explored in this model. BO‐BTs also offer a humanized platform for testing the therapeutic efficacy and side effects on neurons in preclinical trials. We also introduce the BTOs establishment fused with other advanced techniques, such as 3D bioprinting. So far, over 11 brain tumor types of BTOs have been established, especially for glioblastoma. We conclude BTOs could be a reliable model to understand brain tumors and develop targeted therapies.

## 1 BACKGROUND

Brain tumors cause high morbidity and mortality globally and are challenging to treat due to the complexity of the anatomical location and biological characteristics; predictably, the incidence and 5‐year survival rate of malignant brain tumors have not changed considerably in the past decades.^1^ Unfortunately, patients with brain tumors receive minimal benefits from current treatments.^2^ For instance, the median survival period of glioblastoma (GBM) patients has only been extended by 3.7 months on average compared to the 1980s, despite advances in neurosurgical resection, chemotherapy, and radiotherapy.^3^, ^4^, ^5^ Moreover, multiple novel therapies for brain tumors have recently been developed, including targeted therapy, immunotherapy, tumor vaccines, and oncolytic viruses. However, the limited success of these therapeutics has restricted their clinical application prospect.^6^

The key obstacle is the lack of an appropriate model to comprehensively mimic the characteristics of brain tumors, which hampers the investigation of tumor biology and the development of novel therapies and drug screening for precision treatment.^4^ Two‐dimensional cell culture is convenient and represents accurate molecular signatures in the early generations.^7^ However, subsequent generations may show genetic and transcriptional changes owing to spontaneous variations and selection of cells with rapid proliferation.^8^, ^9^ It also loses three‐dimensional functional cell–cell interactions, which further reduces its applicability under in vivo conditions. Tumor spheroids retain the three‐dimensional architecture and physical cell interactions^10^ but consist of tumor cells with limited intra‐tumor heterogeneity and lack a tumor microenvironment (TME).^11^, ^12^ The TME, where stromal interactions, immune responses, and extracellular matrix (ECM) generation occur, plays an important role in tumorigenesis and therapeutic resistance. Brain tumor TMEs include specific cell types, such as neurons, astrocytes, microglia, macrophages, tumor‐infiltrating lymphocytes, vascular cells, and fibroblasts.

Compared with two‐dimensional cultures and spheroids, patient‐derived xenografts (PDXs) can maintain TME. PDXs are generated from surgical tissues transplanted into immunosuppressed rodents and consistently maintain primary tumor phenotypes and heterogeneity.^13^, ^14^ However, species differences at the gross neuroanatomical, cellular, and molecular levels have led to varied results^15^; additionally, low success rate, prolonged latency, and high cost impede the broad application of PDXs. Tumor organotypic explant cultures are established from patient tumors mechanically and preserve the cellular composition and TME as present in situ.^16^ While this model has been applied to investigate tumor invasion and drug responses, it showed short‐term survival and poor expandability.^16^, ^17^, ^18^ The manipulative complexity and the subsequent cellular reaction after mechanical slicing also impeded the application.^19^ Altogether, none of the current models for brain tumors are optimal and technical innovations are required (Table 1).

**TABLE 1:** Characteristics of the three mainstream preclinical cancer models.

Features	2D cell lines	PDXs	Tumor organotypic explant	BTOs
Basic criteria for preclinical model				
Time demand	+++	+	+	++
Success rate	++	+	+	+++
Cost	+++	+	+	++
Technical difficulty	+	+++	+	++
Long‐term stability	+	++	−	+++
Real‐time imaging	+++	+	++	+++
Manipulability	+++	+	+	++
Representation of primary tumor				
Molecular preservation	+	++	+++	+++
Phenotype preservation	+	+++	+++	++
TME preservation	−	+++	+++	++
Application in basic research				
Tumorigenesis	++	+	+	+++
Interactions with TME	−	+++	+++	++
Cancer stem cell	+	++	+	+++
Therapy resistance	+	+	++	+++
Application in precision therapy				
Drug testing	++	+	+	++
Biobank	++	+	−	+++
Preclinical research	+	++	+	+++
Reducing side effects	+	++	+	+++

Abbreviations: +++, best; ++, suitable; +, possible; −, unsuitable.

Organoids are three‐dimensional cellular self‐aggregates that precisely mimic the source tissue and are commonly derived from human pluripotent stem cells (hPSCs) or cancer stem cells (CSCs). The first brain organoids and brain tumor organoids (BTOs) were reported in 2013 and 2016, respectively.^20^, ^21^ Organoids can maintain multiple cellular lineages and preserve complex cell–cell communications.^22^, ^23^ Importantly, this model recapitulates the genotype and phenotype, including the heterogeneity of the parental tumor.^20^, ^24^ Furthermore, organoids provide a humanized TME to investigate brain tumors. Currently, BTOs can be generated within 1–2 weeks with success rates far higher than those of PDX. They can be cultured for long‐term biobank application.^24^ Different forms of BTOs can be customized to research objectives and brain tumor features (Table 2). These advantages of BTOs have attracted attention in preclinical research. In this comprehensive review, we introduce the different forms of established BTOs and their characteristics, including their strengths and applications in the study of CSCs, therapy resistance, drug testing, and preclinical research.

**TABLE 2:** Characteristics of the different forms of BTOs.

Form of BTOs	Advantages	Shortcomings	Suitable application	Current establishment
BTOs derived from patient somatic cells	1. Preserving the intrinsic germline mutations 2. Providing a precious platform for human‐specific, systematic genetic diseases with pathology of brain tumors	1. Long‐time establishment 2. No immune and vascular cells	1. Discovering the biological mechanism and interventable targets of the diseases 2. Monitoring the natural trajectories of tumorigenesis and development	TSC, NF1
BTOs derived from hPSCs with genetic manipulations	1. Modeling tumorigenesis and development Flexibility of the timing to introduce driver mutations 2. Capability to artificially introduce mutations 3. Containing both the tumor and normal cells	1. Long‐time establishment 2. No immune and vascular cells 3. Ignorance of cellular heterogeneity 3. The complexity of genetic manipulation techniques 4. Unknown effects of the artificially introduced genes on their own gene expression	1. Identification of the oncogene and understanding the genetic function 2. Discovering therapeutic targets 3. Studying invasion and cell–cell interaction 4. Investigating susceptibility of brain tumors	GBM, MB, ATRT, CNS‐PNET, NF1
BTOs derived from tumor cells	1. Maintenance of molecular features (genetics, epigenetics, transcriptomics, metabonomics) 2. Maintenance of phenotypes when orthotopically transplant into animals 3. Diversity of niche and cell subtypes 4. Maintenance of intra‐ and inter‐ heterogeneity 5. Maintenance of functional cell–cell interaction among tumor cells 6. Maintenance of partial stromal cells (e.g., immune and vascular cells) 7. Capability to co‐cultured with immune cells directly 8. Similar responses to therapies as original tumors 9. Fast and scalable establishment 10. High success rate for establishment 11. Providing a precious platform for benign or slow‐proliferated brain tumors 12. Biobankability	1. Lacking TME, gradual reduction of stromal cells 2. Limited interactions with non‐tumor cells 3. Gene drift after high passage	1. Drug testing and high‐throughput screening 2. Preclinical studies (targeted drugs, immunotherapy, oncolytic virus) 3. Establishing biobank 4. Discovering the biological mechanism (therapy resistance, cancer stem cells, tumorigenesis)	GBM, LGG, BM, MB, Schwannoma
Brain tumor cells/spheres co‐cultured with brain organoids	1. Containing both the tumor and normal cells 2. Maintenance of the interactions between tumor cells and TME (neurons, astrocytes) 3. Maintenance of phenotypes 4. Closest model to the original tumor (molecular features) 5. Real‐time imaging of invasion	1. Long‐time establishment 2. No immune and vascular cells	1. Studying the mechanism of interactions between tumor cells and TME, such as invasion and tumor‐promoting effect of TME 2. Evaluation of the therapeutic effects on invasiveness 3. Evaluation of the dosage and side effect of therapy	GBM, LGG, Meningioma, BM, MB, ATRT

Abbreviations: ATRT, atypical teratoid rhabdoid tumor; BM, brain metastasis; GBM, glioblastoma; LGG, lower grade glioma; MB, medulloblastoma; NF1, neurofibromatosis type 1; CNS‐PNET, primitive neuroectodermal tumor; TSC, tuberous sclerosis complex.

## 2 NORMAL BRAIN ORGANOIDS

**FIGURE 1:**
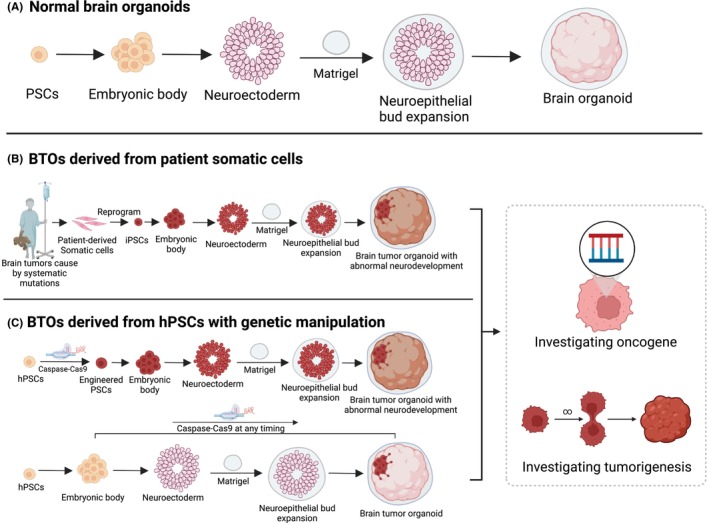
Establishment of BOs and BTOs derived from hPSCs. (A) Protocol of generating normal BOs. (B) Protocol of generating BTOs derived from patient somatic cells. (C) Protocol of BTOs derived from hPSCs with genetic manipulation. Genetic manipulation could be carried on at multiple timepoints during the generation of BTOs to model the role of genetic mutations during neurodevelopment. These two types of BTOs could be applied for investigation of oncogene function and tumorigenesis process. The diagram was created with BioRender.com.

In. 2013, Lancaster et al^21^ established brain organoids derived from embryonic stem cells (ESCs), recapitulating the three‐dimensional structural organization with neural identity and differentiation. The procedure entailed: (1) inducing hPSCs to generate embryoid bodies (EBs); (2) feeding EBs and initiation of germ cells; (3) induction of the neural ectoderm; (4) transfer of neuroepithelial tissues to Matrigel droplets and neuroepithelial bud expansion; (5) brain tissue growth and expansion.^21^, ^25^, ^26^ Using this technique, normal brain organoids can be generated within a month and maintained for more than a year, exhibiting the spatial topography identified by region‐specific markers (Figure 1A).^21^, ^25^, ^26^ To date, different region‐specific brain organoids have been established, including the forebrain, midbrain, hindbrain, choroid plexus, cerebellum, hypothalamus, and pituitary.^27^, ^28^, ^29^, ^30^, ^31^, ^32^, ^33^ Normal brain organoids, which are differentiated and self‐aggregated from hPSCs, also preserve multiple cell types including neuronal and astrocytic sublineages. Oligodendrocytes,^34^ vascular endothelium,^35^ and microglial cells^36^ can be derived using a modified protocol. These features enable normal brain organoids to mimic the human brain to a high degree, and extensively model neural diseases.

## 3 BRAIN TUMOR ORGANOIDS DERIVED FROM PATIENT SOMATIC CELLS

Some types of brain tumors resulting from specific germline mutations emerge and grow during neurodevelopment, but lack an appropriate in vivo or in vitro model.^37^, ^38^ Several studies have collected somatic cells (blood mononuclear cells and fibroblasts) from patients with these tumors, reprogrammed these cells to induced pluripotent stem cells (iPSCs), and generated brain organoids from reprogrammed iPSCs with intrinsic genetic defects. During the growth of this form of brain organoids, brain tumors initiate at a specific developmental point and proliferate within the brain organoids, exhibiting morphological progression, biological behavior, and signaling mimicking human disease (Figure 1B). Such models have been demonstrated in studies of neurofibromatosis (NF) and tuberous sclerosis complex (TSC).^39^, ^40^ However, the ability to simulate the developmental trajectories of these brain tumors driven by germline mutations cannot be supported by other models. Moreover, animal models cannot recapitulate human‐specific neurodevelopmental diseases, and cell lines lack the three‐dimensional cell–cell interactions that are essential for neurodevelopment. Eichmüller et al. generated brain organoids for TSC derived from patients with *TSC2* mutations. The organoid model recapitulated the pathological features of TSC, exhibiting both brain tumors and dysplastic cortical regions. Using scRNA‐seq and extensive histological validation, a specific interneuron progenitor population called the caudal late interneuron progenitor (CLIP) cells was identified, which are dispensable and responsible for the initiation of both tumor and cortical tuber lesions. These cells originated from the caudal ganglionic eminence during mid‐gestation in the fetal brain and were previously unidentified. Excessive CLIP cell proliferation initiates both tumor and brain abnormalities during neurodevelopment, depending on EGFR signaling, indicating a possible therapeutic target.^39^ This organoid technology provides a valuable humanized platform to model these rare genetic diseases.

## 4 BRAIN TUMOR ORGANOIDS DERIVED FROM hPSCs WITH GENETIC MANIPULATION

In addition to BTOs established from patient‐derived iPSCs with inherent genetic defects, BTOs can also be established from PSCs (including iPSCs and ESCs) by introducing oncogene overexpression or loss of tumor‐suppressor gene function at different developmental stages (Figure 1C). This form of BTOs can be used to investigate the role of genetic mutations in tumorigenesis and tumor development (Table 3). EGFRvIII is a common mutation in GBMs. EGFRvIII was introduced into ESCs to generate EGFRvIII^OE^ organoids, which exhibit excessive cell proliferation and astrogenesis at the expense of neurogenesis, similar to that observed during GBM pathogenesis. At the EBs stage, neural stem and progenitor cells, which are considered the origin of many different brain tumors, expand on the surface of the EBs.^41^, ^42^, ^43^ Bian et al^44^ introduced plasmids containing oncogene‐amplifying and tumor‐suppressing mutations into EBs via electroporation at the end of neural induction culture, prior to Matrigel embedding. They modeled the formation of GBM‐like and primitive neuroectodermal tumor (CNS‐PNET)‐like tumors, which induce tumor overgrowth and showed similar transcriptomic signatures. CNS‐PNET is a rare and malignant brain tumor that lacks in vivo and in vitro models, necessitating the use of organoid technology to further investigate this rare tumor type. At the brain organoid expansion stage, brain organoids do not achieve complete postmitotic maturity and never completely lose their neural stem cell population.^25^ GBM^45^ and medulloblastoma^46^ organoids were established using oncogene electroporation of brain organoids in matrigel. Because only a small proportion of the cells in brain organoids are genetically engineered, these BTOs partly mimic human tumorigenesis because they contain both tumor and normal tissues. This allows for the study of interactions between tumors and normal cells, and their invasiveness. Another potential advantage of this model is that organoids established from iPSCs derived from patients or susceptible populations can be further used to test the susceptibility of individuals to different combinations of driver mutations, thereby meeting the needs of precision oncology.^44^

**TABLE 3:** Summary of tumorigenesis studies using BTOs.

Brain tumor type	Driver gene	Stage of mutations occurring	Findings	References
Glioma	*EGFRvIII* ^*OE*^	hESCs	Excessive gliogenesis at the expense of neurogenesis	^138^
	1.*CDKN2A* ^*−/−*^ */CDKN2B* ^*−/−*^ */EGFR* ^*OE*^ */EGFRvIII* ^*OE*^ *2.NF1* ^−/−^/*PTEN* ^−/−^/*TP53* ^−/−^ (*p53* ^−/−^) *3.EGFRvIII*OE/*CDKN2A* ^−/−^/*PTEN* ^−/−^	End of neural induction culture	1. Inducing tumor over‐proliferation and invasion in organoids 2. Similar transcriptome as GBM 3. Exhibition of distinct cellular identity 4. Viability and invasion when transplant in vivo; 5. Identification of interactions between tumor and normal cells; suitability for targeted drug testing	^44^
	*TP53* ^−/−^ */PTEN* ^−/−^ */MEOX2* ^*OE*^	Cerebral organoids expanding	MEOX2 cooperated with p53 and PTEN loss to induce excessive proliferation	^139^
	*TP53* ^−/−^/*HRas* ^*G12V OE*^	Cerebral organoids expanding	1. Inducing tumor over‐proliferation and invasion in organoids 2. Similar transcriptome as GBM 3. Tumorigenesis and invasion when transplant in vivo 4. Serial transplantability	^45^
Medulloblastoma	*Otx2* ^*OE*^ */c‐MYC* ^*OE*^	Cerebellar organoids expanding	1. Inducing over‐proliferation of cerebellar progenitor cells and impairing their differentiation 2. Similar cellular identity and methylation profile as medulloblastoma 3. Identification of SMARCA4 and EZH2 as therapeutic targets	^46^
CNS‐PNET	*MYC* ^*OE*^	End of neural induction culture	1. Inducing tumor proliferation 2. Similar transcriptome as CNS‐PNET 3. Exhibition of distinct cellular identity 4. Proliferation and exhibition of characteristic pathological features when transplant in vivo	^44^
ATRT	*SMARCB1* ^*−/−*^	hiPSCs During neuronal differentiation	Inducing defects in neuron formation 1. Inducing instability among neural progenitors and failure in neural maturation which contribute to tumorigenesis 2. Similar transcriptome as ATRT	^56^
NF1	*NF1*	hiPSCs	Differential effects of NF1 mutations on cerebral organoid neural progenitor cells proliferation, apoptosis, and differentiation	^40^
TSC	TSC^*+/−*^	hiPSCs	1. Recapitulating the emergence of both brain tumors and dysplastic cortical regions during organoids development 2. Identification of a specific interneuron progenitor population (CLIP cells) which result in both tumor and cortical tuber lesions 3. Over‐proliferation of CLIP cells depending on EGFR signaling, suggesting a therapeutic target	^39^

Brain tumors have been suggested to arise from or be driven by neural stem‐like cells.^47^, ^48^, ^49^, ^50^, ^51^ Recurrent mutations in brain tumors also affect neurodevelopment.^52^, ^53^ Similarly, some perturbed signaling pathways in neurodevelopment lead to the initiation and proliferation of brain tumors.^54^, ^55^ Therefore, tumorigenesis may be closely associated with neurodevelopment. Brain organoid growth mimics neurodevelopment and contains multiple cellular lineages in the human brain. By performing genetic manipulation at different stages of organoid establishment, brain organoids can be developed as an optimal model to study tumorigenesis, especially for pediatric brain tumors that appear during active neurodevelopment. For example, atypical teratoid rhabdoid tumors (ATRTs) are challenging pediatric brain cancers caused by the inactivation of *SMARCB1* during neurodevelopment. During neuronal differentiation in brain organoids, *SMARCB1* was knocked down using CRISPR/Cas9. The *SMARCB1* ^−/−^organoids exhibited a transcriptomic profile similar to that of ATRTs and demonstrated instability among neural progenitors and failure in neural maturation, contributing to tumorigenesis.^56^

## 5 BRAIN TUMOROIDS

**FIGURE 2:**
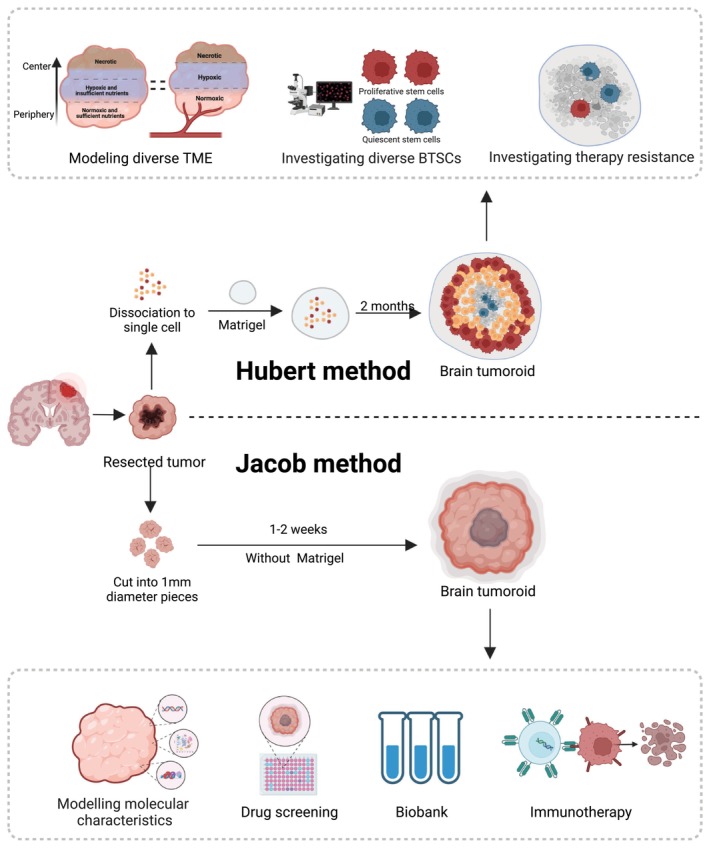
Establishment of brain tumoroids and their applications. Hubert brain tumoroids are generated within matrigel as BOs, composed of multiple TME due to gradient exposure to nutrients and oxygen. Jacob brain tumoroids could be generated within 2 weeks without cell–cell dissociation and matrigel, which are the most clinically applicable BTOs. The diagram was created with BioRender.com.

Based on the tumoral property of infinite proliferation, the models of brain tumors can be generated from brain tumor specimens, such as immortalized tumor cell lines, patient samples, xenografts, and genetically engineered glioma models. In 2016, Hubert et al. established brain tumoroids directly from GBM specimens. They dissociated samples derived from patient tumors into single cells and embedded approximately 1000 suspended cells per organoid into Matrigel (Figure 2). GBM tumoroids (GBOs) expanded prolifically to sizes of 3–4 mm in 2 months, demonstrating reduced growth, stability, and viability for more than a year without passaging.^20^ When dissociated into single cells and implanted into mouse brains, these GBOs could maintain invasiveness, while GBM tumor spheres lost their invasive phenotype, indicating that cell growth conditions may help maintain the phenotype. The greatest strength of Hubert GBOs is their ability to recapitulate cellular diversity and the TME. Because GBOs do not have a vascular system, a gradient was observed resulting from exposure to growth‐supporting materials (oxygen, exogenous growth factors, nutrients) from the outer zone to the core of the GBOs, resulting in microenvironmental variation. The outer zone of Hubert GBOs modeled the perivascular niche with sufficient growth‐supporting materials and exhibited rapid proliferation. The inner zone modeled the perinecrotic niche far from the vasculature and exhibited hypoxic, quiescent, and even necrotic properties. The TME exerts considerable effects on tumor cells, including GSCs. In Hubert GBOs, GSCs were distributed more densely and proliferated faster in the outer zone, but were sparse and tended toward quiescence in the inner zone. Importantly, this model spontaneously contained different states of GSCs, providing an experimental platform for studying their biological characteristics. For example, GSCs could transform into quiescent state under chemotherapy to develop resistance and keep living with a possibility to recur.^57^ However, currently the models for quiescent GSCs are lacking owing to rapidly proliferating populations. Hubert GBOs may help to solve this dilemma and allow the identification of quiescent GSC markers and targets.

Metabolism is a key facet of glioma growth and metastasis, and cannot be accurately represented outside the influence of the TME. The diverse microenvironment in Hubert GBOs also triggers metabolic alterations that can serve as therapeutic targets. For example, hypoxia can induce lipid droplet biogenesis to protect cells from oxidative damage and provide energy.^58^ Lipid enrichment has been identified in hypoxic GBOs. Because GSCs are mainly distributed in the outer zone, they have low lipid droplet accumulation compared to non‐GSCs. Deeper lipidomic analysis showed that GSCs contained reduced levels of major classes of neutral lipids, but displayed higher polyunsaturated fatty acid production compared to non‐CSCs, due to high expression of fatty acid desaturase (FADS1/2). Upon knocking down FADS1/2, the viability and self‐renewal ability of GSCs are damaged, indicating a therapeutic target.^59^ Therefore, Hubert GBOs provide a platform to identify the abnormal metabolisms and target them.

In Hubert GBOs, different cells exhibited different responses to therapies and more resistance compared to cell lines and tumorspheres (Table 4).^20^ By using the techniques of cellular tracking and sorting, specific subpopulation of GBM cells could be identified from GBOs to investigate.^60^, ^61^ For example, by isolating quiescent GBM cells, bioinformatics analyses and functional assays showed that hypoxia and TGFβ signaling may drive the identity of quiescent GBM cells, providing a potential mechanism to ameliorate therapy resistance.^60^ While targeting populations of resistant cells is promising, complicated cell–cell crosstalk within GBOs could also be a potential mechanism supporting therapy resistance. Tunneling nanotubes (TNTs) and tumor microtubes (TMs) have both been found in Hubert GBOs, of which TMs have not been previously identified in vitro. TMs are membranous extensions that allow ion flux through GAP‐junctional proteins, providing rapid neurite‐like communication between cells. TNTs are membranous structures that are open at both extremities, allowing cytoplasmic continuity and transportation of organelles such as mitochondria between connected cells.^62^ Due to this transfer, tumor cells acquire new abilities such as metabolic plasticity and treatment resistence.^62^, ^63^, ^64^ In GBOs, mitochondrial transfer through a functional TNT connection was observed among tumor cells. TMs cooperate with TNTs to participate in therapy resistance.^65^ Mitochondrial transfer between tumor cells can provide metabolic support and rescue aerobic respiration for recipient tumor cells in response to treatment‐related stress, revealing a partial mechanism of therapy resistance in patients.^64^ Therefore, glioblastoma cells in GBOs may overcome therapy through cooperation in the TME, aided by complicated cellular connections, which may be closer to the responses observed in parental tumors and an appropriate model to study therapy resistance.

**TABLE 4:** Different therapy responses of preclinical in vitro models.

Therapy	2D	Tumorsphere	GBOs	References
TMZ	+	+	−	61, 93
Radiation	+	+	−	61, 93
TMZ + Radiation (Stupp)	+	+	−	61, 140
Vismodegib	+		−	^80^
Vismodegib+Stupp	+		−	^80^
Disulfiram	+		−	^80^
Disulfiram+Stupp	+		−	^80^
Omipalisib	+		−	^80^
Omipalisib+Stupp	+		−	^80^
Parthenolide+Stupp	+		−	^80^
Compound JVM‐3‐55		+	−	^141^
Compound PNR‐5‐88		+	−	^141^
Compound PNR‐7‐84		+	−	^141^
Ruxolitinib		+	−	^61^
Ibrutinib		+	−	^61^
ruxolitinib		+	−	^61^

Abbreviations: +, sensitive; −, resistant.

In 2020, Jacob et al reported a revolutionary method that could generate GBM tumors directly from resected GBM samples rather than via dissociation, retaining native cell–cell interactions (Figure 2). By optimizing a chemically defined medium, they cultured tumoroids with few exogenous growth factors and no Matrigel to minimize clonal selection and decrease potential treatment confounders. Besides, growing without matrigel, which is a kind of undefined and complex ECM, could also avoid the unstability and matrigel‐specific effects.^66^ Tumoroids derived from patients without dissociation also retained a heterogeneous cellular composition, including immune and endothelial cells. In this model, immune and endothelial cells can persist for more than 8 months and gradually decrease over time. Moreover, this method generated GBM organoids approximately 1–2 weeks after initial surgical resection with high fidelity and an overall success rate of 91.4%.^24^, ^67^ Jacob GBOs depend on the gradient exposure of growth‐supporting materials and precisely recapitulate the intra‐ and inter‐tumoral heterogeneity from genotype to phenotype. Profiling of somatic variants and copy number variants (CNV) in GBOs is largely similar to tumors derived from different patients, indicating inter‐tumoral heterogeneity. The GBOs derived using this method from different subregions in the same patient also showed subregion‐specific mutations.^24^ Specifically, Jacob GBOs preserved EGFR mutation, a driver in GBM, which was rapidly lost in two‐dimensional culture.^67^

GBOs also showed high similarity to parental GBM samples at the transcriptome level for over 12 weeks. Even for the macrophage/microgial‐related genes, the expression was comparable between GBOs and parental tumors for 2 weeks. Due to the disability of replication and immortality for non‐tumor cells, the most differentially downregulated genes between GBOs and parental tumors were immune‐ and blood‐related genes, indicating incomplete retention of immune cells and blood cells over a long period of time relative to in vivo conditions. Furthermore, scRNA‐seq analysis showed that cellular and molecular signatures in GBOs were highly similar to those of the parental tumor, maintaining cell‐type heterogeneity and molecular properties.^24^ Finally, the GBOs preserved similar morphology compared to parental tumors and could be transplanted into the mouse brain intact, displaying not only invasiveness, but also angiogenesis.

The omics revolution has led to the identification of various targets and a more comprehensive view of the molecular signaling underlying brain tumors through the integration of genomic, epigenomic, transcriptomic, metabolomic, and proteomics data.^68^, ^69^, ^70^ The classification of patients based on multi‐omics profiling enhances precise diagnosis and therapy. However, intratumoral heterogeneity and limited amounts of tumor material available for omics analysis may hamper these advances.^71^, ^72^, ^73^ The extensibility and precision modeling offered by GBOs provide a platform for solving this dilemma. To establish a living biobank for storing omics information, Jacob et al optimized the procedures to cryopreserve GBOs long‐term by: (1) cutting GBM tumoroids into small pieces, (2) pre‐incubating them in freezing medium to allow complete perfusion before freezing, and (3) incubating GBM tumoroids with the ROCK inhibitor before freezing and during thawing to inhibit cell death.^67^, ^74^ After recovering these GBOs from the cryopreserved state, they were capable of maintaining their characteristics and growth. GBOs expanding exponentially on serial passage are generally deemed biobankable, which means that the current tumoroids for all brain tumor types are potentially useful for this purpose.^75^ Importantly, the establishment of biobanks guarantees reproducibility.^76^

Because of the rapid establishment and precise recapitulation of parental tumors, tumoroids have been applied in drug testing and could potentially be used to select efficacious therapies for individual patients. At the whole cancer level, patient‐derived tumor organoids accurately predicted patient responses to therapy with 81% sensitivity and 74% specificity.^77^ For GBM, in an observational study, Jacob et al. reported that the responses of GBOs were consistent with those of patients, with 83% sensitivity and 88% specificity.^24^ Loong et al^78^ used GBOs to prospectively screen drugs for patients to identify targetable mutations using genetic sequencing, which finally selected everolimus and achieved a real effect in the patient. Currently, many studies have used GBOs to test drugs (Table 5). Because treatment for brain tumors, especially malignant brain tumors such as GBM is time‐constrained, GBOs are the most frequently used BTOs for drug testing. Owing to the manipulability of GBOs, several techniques have been incorporated to quicken and scale up drug testing, such as 3D bioprinting and microarray establishment. Importantly, the process of generating GBOs for drug testing can be completed within a month, possibly enabling clinical management and high‐throughput drug screening. Similar to the results of Loong et al,^24^, ^79^ many studies have reported that the responses of GBOs to targeted drugs were not consistent with targeted mutations, confirming that mutation analysis alone without functional testing is insufficient to predict response to treatment. GBOs could also provide a platform to study the biological mechanisms of novel effective drugs that have not been reported in GBM. For example, the proteasome inhibitor carfilzomib was identified as a targeted drug from high‐throughput screening of 320 drugs combined with proteomic and bioinformatic analyses and a series of functional assays in GBOs.^80^ GBOs can also be used to predict the effects of combination therapy, which can improve outcomes in patients with malignancies.^81^ The responses of GBOs to combination therapy showed greater effects than those of monotherapy.^82^

**TABLE 5:** Summary of drug testing studies using GBOs.

Treatment	Target	Cases	Consistency with mutations	Generation time	Treating time	Combined methods	Indicator	References
TMZ Dianhydrogalactitol Geftinib Erlotinib AZD3759 AG490 Daphtenin Abemaciclib Palbociclib 42 FDA‐approved drugs	Alkylating agent Bi‐functional alkylating agent EGFR/ErbB inhibitor EGFR/ErbB inhibitor EGFR/ErbB inhibitor EGFR/ErbB inhibitor EGFR/ErbB inhibitor CDK4/6 inhibitor CDK4/6 inhibitor Various	18 18 16 16 16 16 16 16 16 1	Yes Yes Yes Yes Yes Yes Yes Yes Yes Yes	3 days	1 week	1.3D bioprinting 2.GBOs derived from mice transplanted with GBOs	High content imaging system recognizing viable cells	^142^
TMZ + Radiation (Stupp) Gefitinib Trametinib Everolimus	Standard therapy EGFR inhibitors MEK inhibitor mTOR inhibitor	8 10 4 3	No Yes Yes No	1–2 weeks	1 week	None	Immunohistology of Ki67	^24^
TMZ BEZ235 Niraparib TMZ + BEZ235 Niraparib + BEZ235	Alkylating agent PI3K/mTOR inhibitor PARP inhibitor Combined therapy Combined therapy	2	– – Yes – Yes	1–2 weeks	3 days	4D bioprinting	1. Intracellular ATP cell viability assays (no detailed description) 2. Live cell imaging and Realtime apoptosis by measuring activated caspase 3 3. Immunofluorescence of GFAP, BMI1, pS6	^82^
Dacomitinib NSC59984	EGFR inhibitors p53 pathway activator	2	–	1 week	3 days	3D bioprinting	Intracellular ATP cell viability assays by Celltiter Glo	^126^
Compound JVM‐3‐55 Compound PNR‐5‐88 Compound PNR‐7‐84	NFk‐B inhibitor COX‐2 inhibitor Tubulin inhibitor	3	–	15 days	1, 2, 3, 6, 8, 15 days	Select drugs from 22 drugs by cytotoxicity and invasion effects on tumorspheres	Automatic microscopic measurement of the area of cells invading into the matrix	^142^
Stupp Vismodegib w/wo Stupp Disulfiram w/wo Stupp Omipalisib w/wo Stupp Costunolide w/wo Stupp Parthenolide w/wo Stupp	Standard therapy Hh/GLI inhibitor ALDH inhibitor PI3K/mTOR inhibitor hTERT inhibitor HDAC/IKK‐β/NF‐κB inhibitor	1	–	10 weeks	10 days	Select drugs from 65 drugs by cytotoxicity effects on 2D cells	Intracellular ATP cell viability assays (no detailed description)	^140^
Everolimus Cobimetinib Erlotinib Vemurafenib TMZ EPZ015666	mTOR inhibitor MEK inhibitor EGFR/ErbB inhibitor Raf inhibitor Alkylating agent PRMT5 inhibitor	1	Yes Yes Yes Yes No No	–	–	Select drugs based on target capture sequencing	Cytotoxic sensitivity (no detailed description)	^78^
Carfilzomib	Proteasome inhibitor	2	Yes	–	2 or 3 days	Select carfilzomib from 320 drugs by cytotoxicity effects on tumorspheres and analyses of drug dose–response curve	Immunofluorescence of cleaved caspase 3 and CD133	^80^
Temozolomide Ibrutinib Lomustine Ruxolitinib	Alkylating agent JAK/STAT3 inhibitor JAK/STAT3 inhibitor JAK1/2 inhibitor	6	–	–	6 days	Creation of organoid microarrays	1. Intracellular ATP cell viability assays by Celltiter Glo 2. DNA content analysis 3. Immunofluorescence of cleaved caspase 3 and SOX2 4. Immunohistochemistry staining	^61^

While immunotherapy has achieved great success in several types of cancer, its efficacy on brain tumors is limited.^83^ Current in vitro models for brain tumors often lack cellular heterogeneity and do not maintain mutations that lead to altered surface antigens, which hampers preclinical studies of immunotherapies. Because Jacob GBO retains immune cells, they can serve as in vitro model to develop immunotherapies. For example. inhibiting HSPA7, an immune‐related pseudogene, increased the efficiency of anti‐PD1 therapy in GBOs by reducing macrophage infiltration and shifting the TME from an immunosuppressive state to an immune‐activated state.^84^ Chimeric Antigen Receptor T (CAR‐T) cells could also be co‐cultured with GBOs to test the efficacy and specificity. Using the co‐culturing system, 2173 CAR‐T cells targeting EGFRvIII^+^ cells were found to specifically kill the targeted cells, but retain EGFRvIII^−^ cells in GBOs.^24^, ^67^ In the future, more syngeneic immune cells and tumor‐infiltrated lymphocytes can be sorted and added to BTO co‐culture systems to promote the development of immunotherapy technologies against brain tumors.

Tumor‐treating fields (TTFields) have achieved great efforts for GBM patients in clinical trials. However, patients are also possible to resist to TTFields and it is still unknown which patients are likely to maximally benefit from TTFields. Besides, the mechanisms of TTFields resistance are undiscovered. Using the patient‐derived GBOs, Nickl et al^85^ observed different responses to TTFields and found a TTFields‐resistant GBO. The fast establishment and the close representation make GBOs suitable for screening patients sensitive to TTFields. Meanwhile, GBOs were also capable of further investigating the mechanism of TTFields resistance and find the target to sensitize the efficacy. However, the accuracy for the response to TTFields between GBOs and patients is needed to be confirmed in future studies.

Although GBM is the most common malignant tumor source of brain tumors, many other brain tumoroid models have been established. Organoid technology can be used to establish models of benign or slowly proliferating brain tumors, which have limited experimental models or are difficult to culture in vitro. For example, in vitro models for lower grade gliomas (LGGs) are hard to generate; interestingly, they have been established as tumoroids in 4 weeks with an 87% success rate and could be maintained for months by modifying the Jacob method and using lower oxygen conditions (5%) during culture, probably due to the activated HIF2α under 5% oxygen. The established LGG tumors not only presented the same histology, stem cell markers (SOX2), proliferation (Ki67), vascular composition (CD31), macrophages/microglia (Iba1), and genetic alterations as parental tumors, but also showed similar metabolomics. Oncoprotein IDH enzymes were observed pervasively using immunohistochemistry. More importantly, 2HG accumulation was observed in LGG tumors, comparable to parental tumors, using liquid chromatography‐mass spectrometry analysis.^86^

Meningioma is the most common primary tumor of the brain and is derived from the neural crest.^1^ Meningiomas have a high proportion of interstitial matrix; therefore, dissociating the original tumor samples using enzymatic methods is hard to perform without disrupting cell viability. Most meningiomas are benign and proliferate slowly. This has led to a lack of models for this disease.^87^ Organoid techniques can be used to establish meningioma tumoroids by embedding the dissociated meningioma cells into matrigel with the supplementation of growth factors similar to the generation of cerebral organoids.^88^ The establishment was within 2 weeks with 100% success rates, recapitulating multiple characteristics of the parental tumors.^88^ Molecular features, genetic mutations, chromosome structure, DNA methylation, and RNA expression were all maintained in meningioma tumoroids from parental tumor tissues.^88^ These meningioma tumoroids showed histological and morphological features similar to those of the parental meningiomas of different grades. Meningioma markers and low proliferative features were also observed in meningioma tumoroids.^87^, ^88^ FOXM1 expression is correlated with increased proliferation in meningiomas, and inhibiting FOXM1 using thiostrepton combined with radiotherapy could efficiently kill tumor cells, indicating a novel targeted therapy.^88^

Other brain tumors, including medulloblastoma (MB),^89^ brain metastases,^20^ and schwannoma^90^ tumoroids, have also been established and used for scientific exploration. For example, in MB tumors, the proteasome inhibitor NPI‐0052 combined with γ‐radiation showed synergistic apoptotic effects on MB cells.^89^ However, these tumoroids were still established based on the matrigel and multiple supplements supporting growth, more optimized approaches were encouraged.

## 6 BRAIN ORGANOIDS CO‐CULTURED WITH BRAIN TUMORS (BO‐BT)

**FIGURE 3:**
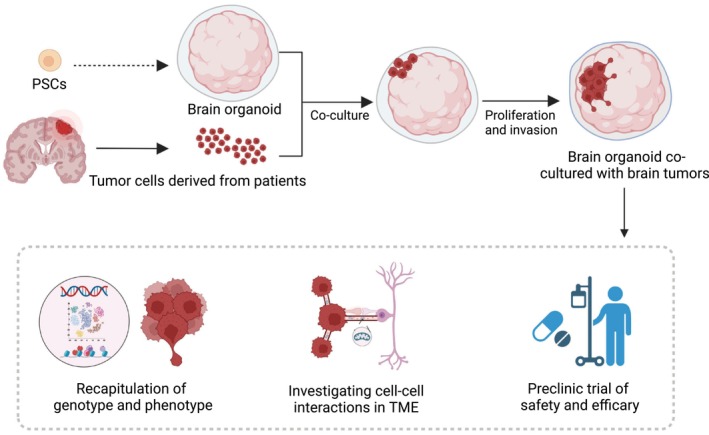
Establishment of brain organoids co‐cultured with brain tumors and their applications. BO‐BTs are the most similar BTOs as original tumors, which could model humanized interactions between tumor cells and brain components in vitro. The diagram was created with BioRender.com.

To study tumor invasion and cell–cell interactions between tumors and normal cells, brain organoids can be co‐cultured with brain tumor cells/spheres (Figure 3). CSCs are a group of cells that most recapitulate tumors molecularly and phenotypically and are most commonly co‐cultured with organoids.^45^, ^91^, ^92^ Depending on the tissue‐clearing method and microscopy technique used, invasive protrusions, and microtube networks formed in brain tumor cells can be observed and measured as surrogates of invasive ability. Reporter genes, such as luciferase, can also be ectopically expressed in brain tumor cells for real‐time live imaging. Three types of co‐culture patterns were established. First, co‐cultured brain tumor cells with iPSCs and then induced brain tumor organoids. In this pattern, recurrent GBM stem cells (GSCs) exhibited enhanced invasiveness compared to primary GSCs at an early stage. However, both recurrent and primary GSCs stopped growing after day 10 and survived for up to day 20. Second, brain tumor cells were implanted into the established brain organoids. Distinguishing invasiveness between recurrent and primary GSCs was also significant. The invasive protrusions and microtube‐like structures of surgical GBM tumor specimens resembled GSCs formed in this pattern. Third, brain tumorspheres were co‐cultured with established brain organoids. In this system, individual GSCs invade brain organoids, showing a profound tropism of GSCs to the brain tissue. While GSCs were compact in spheres, invasive protrusions, and microtubes could not be quantified.

The generation of GBM cells co‐cultured with cerebral organoids (GLICOs) often takes over a month, which is longer than that for GBOs. However, GLICOs are reported to be the most accurate models when compared with the two‐dimensional, GBOs, and PDX models. Among these models, GLICOs exhibited the highest correlation with parental tumors at many levels, such as similar transcriptomes, diversity of cellular states, and strong stemness and invasiveness signatures.^91^ GSCs in GLICOs also preserved key genetic and signaling components of the parental tumors,^93^ in addition to pathological features and progression capacity. In one study, GLICO models identified aggressive infiltration of tumor cells into cerebral organoids, a pathological feature of grade IV GBM, in a patient with grade II astrocytoma. After 18 months, the second diagnosis of recurrent tumors in the same patient revealed a pathological advancement to grade IV GBM.^79^ These advantages may result from the existence of a suitable TME from brain organoids that contain neurons, axons, myelinated axons, and dendritic synapses. GSCs in GLICOs exhibited reduced apoptosis and markedly enhanced proliferation and tumor microtubes compared to GSCs in GBO, which may also be due to the TME. The interactions of brain tumor cells with TME components and how they affect tumor growth and behavior are now gradually being revealed, although the specific mechanisms remain unknown.^94^

Brain organoids provide a platform for studying the mechanism of interaction between the brain and brain tumors. Compared with PDX, which also contains a TME, brain organoids are humanized, manipulable, and fast for the establishment with a higher success rate, allowing real‐time imaging. scRNA‐seq analysis of GBM cells before and after co‐culture with brain organoids showed that GBM cells could sense the neuron once co‐cultured and upregulate the gene expression related to dispersion and ligand‐receptor interaction between GBM and organoid cells. Therefore, targeting and breaking the cell–cell connections could be a novel therapeutic strategy.^95^ TMs and TNTs have been observed in GLICOs. Their connections with normal cells in brain organoids may be the reason for the enhancement because tumor growth in the brain has been shown to require neighboring cellular activity.^96^ TMs can form synapses with neurons and astrocytes and drive tumor progression in primary brain tumors^97^, ^98^ or brain metastases.^99^ In GLICOs, TMs are found in an interconnected network that can effectively propagate calcium signals for cellular communication. They deeply penetrate the brain organoids and provide potential routes for invasion, proliferation, and interconnection over long distances.^100^ In brain metastases co‐cultured with brain organoids, astrocytes also form GAP junctions with metastatic lung cancer cells, which promotes tumor growth.^101^, ^102^ TNTs were observed in GLICOs under electron microscopy, and cytoplasmic fusions were formed not only between neighboring tumor cells, but also between neurons and tumor cells. Multiple organelle transfers, including mitochondria, were observed between the cell nuclei. Using organoid techniques, tumor cells can form functional TMs and TNTs, thus cooperating with neighboring tumor cells, neurons, and astrocytes in the brain organoids. Tumor cells acted as a synergistic community in organoids, comparable to in situ conditions. Once GLICOs were transferred into two‐dimensional culture conditions, breaking the three‐dimensional cell–cell interaction, the GBM cells exhibited transcriptomes similar to the tumor cells in two‐dimensional culture conditions, downregulation of several genes related to tumor stemness such as SOX4, NFIA, and BCAN, and lost diversity in cellular subtypes. This evidence suggests that the ability of BO‐BT to model parental tumors can be attributed to cell–cell interactions in the TME.

Invasiveness is linked to cancer‐TME crosstalk. By changing the characteristics of brain organoids, the consequent invasive capacity can be altered, thus offering an opportunity for a deeper understanding of the invasive process of brain tumors and discovery of potential therapeutic targets. For example, GSCs exhibited faster and deeper invasion in mature brain organoids compared to younger ones; this was linked with the synaptic protein Neuroligin‐3, which is generated by mature neurons only. By blocking Neuroligin‐3 function, the invasiveness of GSCs was markedly reduced, indicating a potential target. More variants can be changed in brain organoids to further study the interactions between the brain and brain tumors, such as different brain regions or brain organoids derived from syngeneic and non‐syngeneic iPSCs.

Patient‐derived meningioma cells also exhibited phenocopy invasiveness when co‐cultured with brain organoids. In brain organoids, higher grades of meningioma cells exhibited an invasive phenotype, and lower grades of meningioma cells only formed tumorspheres at the surface of the brain organoids. Meningioma cells co‐cultured with brain organoids showed the greatest number of overlapping genes with parental tumors when compared to two‐ and three‐dimensional monocultures. CDH2 and PTPRZ1 have been identified as oncogenes driving the tumorigenesis of meningiomas in brain organoids, indicating potential targets.^103^

With the incorporation of brain components in organoids, GSCs exhibit resistance to chemotherapy and radiotherapy compared with the cells in two‐dimensional culture.^93^ This means that the BO‐BT model can also serve as a model for studying therapy resistance and is even more suitable than brain tumors because of the preservation of TME. The tumor cells in BO‐BT are more sensitive to therapy than cell lines, including GBM treated with TMZ^79^ and NSCLC brain metastases treated with Gefitinib.^103^ However, the reason remains unknown, and though tumor cells in two‐dimensional culture are speculated to be restricted in terms of growth and malignant behavior and thus protected from chemotherapy or radiation therapy, further investigation is required.^79^

GLICOs can be used to evaluate the efficacy of novel therapies in preclinical trials, and have been widely used for this purpose, including the evaluation of cytotoxicity, invasion inhibition, and radiotherapy sensitization. For example, doxycycline is a Nek2‐KD inducer that can activate Nek2 to induce ciliogenesis, thereby causing GSCs to differentiate. Among GLICOs, GSCs exhibit significantly decreased invasiveness caused by doxycycline‐induced differentiation, paving the way for GSC‐targeted therapy. Moreover, GLICOs offer a humanized platform close to the parental tumors in situ because certain therapeutic targets do not exist in animal hosts, such as some long non‐coding RNAs (lncRNAs). Inhibiting a certain primate‐conserved lncRNA screened by the CRISPR interference technique in GLICOs showed decreased tumor growth and stronger radiotherapy effects, which could not be modeled in PDX.^104^

Oncolytic viruses are emerging antitumor therapies that selectively target, internalize, and kill tumor cells while sparing normal cells.^105^ The Zika virus can enter the brain, and this viral infection can lead to neonatal microcephaly and other neurodevelopmental defects; infected adults are often asymptomatic.^106^ Recently, Zika virus was engineered as an oncolytic virus for patients with brain tumors by targeting SOX2 cells.^107^, ^108^, ^109^ Normally, SOX2 is a transcription factor that is expressed at high levels during human neurodevelopment and contributes to the induction of pluripotency.^110^ SOX2 is also highly expressed in many brain tumor stem cells (BTSCs) such as GBM and medulloblastoma.^111^ Using humanized BT‐BO, the effects of oncolytic viruses on both tumor cells and normal cells could be evaluated. In BT‐BO, Zika virus preferentially and effectively infected and killed BTSCs, including GBM, ATRTs, and medulloblastoma, but had limited effects on mature brain organoid size.^107^, ^108^, ^109^ In the future, more oncoloytic viruses that can enter the brain may be engineered to target brain tumor cells, and BTOs represent a major opportunity for preclinical studies of this emerging treatment modality.

The BO‐BT models can also be used to evaluate the safety and tolerance of novel therapies. The neurological impairment caused by tumors and associated therapies can have serious lifelong consequences on daily function and deeply influence the quality of life, including fatigue, memory loss, emotional distress, and sleep disorders. Minimizing the side effects of therapies is as equally important as inhibiting the tumor. The identification of the effective and tolerable range of dosage and therapeutic intensity is important in preclinical trials.^112^, ^113^ Brain organoids as a “mini brain” can be used as a surrogate to evaluate the side effects of antitumor therapy and provide valuable information for clinical decisions. For example, TTFields showed inhibitory effects on GBM cell proliferation at both 75% and 100% duty cycles; the neurotoxicity of brain organoids at 75% was less prominent than at 100%, indicating that 75% may be a better choice.^114^ The targeted drug UM‐002 employed in GLICO showed that higher concentrations (>500 nM) reduced GBM cell proliferation but also induced toxicity in normal brain organoids. In a dose–response study, 100 nM was found to not only be cytotoxic for GBM cells, but also safe for brain organoids.^115^ The neural side effects of radiation^116^, ^117^ and Zika virus^62^, ^63^ have also been evaluated in brain organoids. In addition, normal organoids of other organs that are frequently impaired in systemic therapy, such as the heart, liver, and stomach, can be used to test toxicity.^118^, ^119^ Notably, multi‐organ organoids with tumoroids have been constructed into a connected system with circulation using microfluidic techniques to synchronously test toxicity and treatment efficacy.^119^

## 7 BTO ESTABLISHMENT USING OTHER ADVANCED TECHNIQUES

Internal hypoxia and cell death due to insufficient diffusion of culture media and oxygen are prominent causes for current brain organoid culture methods generating insufficient numbers of mature neurons. However, organotypic slices can bypass diffusion limitations to prevent cell death and enhance neuronal maturation and viability.^120^, ^121^ Sliced brain organoids co‐cultured with brain tumor cells/spheres have been identified as a feasible method for assessing how mature neurons interact with brain tumors. For example, network structures comparable to those of synapses between neurons and GSCs have been observed in this system.^92^ Other types of cells, including microglia, astrocytes, immune cells, and molecules/drugs, can be added to the culture system of organotypic slices to enrich the microenvironment and evaluate the tumor response. The limitations of this method include its relatively short‐term maintenance ability in culture, which lasts for only several weeks, and lack of scalability.^121^

Another deficiency of BTOs is the lack of stromal cells such as immune and vascular cells. Although the Jacob method can retain some of these populations, their presence varies across GBOs because of the heterogeneous occurrence of stromal cells among different regions in tumors and different patients.^24^ Before Jacob's work, BTOs were embedded into an ECM, such as Matrigel. There are three methods to coculture stromal cells with organoids: (1) submerged Matrigel culture, (2) air‐liquid interface (ALI) culture, and (3) microfluidic 3D culture.^122^ Using the submerged Matrigel culture method, astrocytes, and microglia were co‐cultured with brain tumor cells and organoids underneath the culture medium.^103^, ^123^ ALI systems enable more efficient oxygen transportation to sustain the growth of hybrids comprising multiple types of cells.^124^ Using the ALI method, immune cells, and fibroblasts were successfully co‐cultured with meningioma and schwannoma tumors, while oligodendroglioma and GBM failed, perhaps due to relatively smaller samples. Endogenous and syngeneic tumor‐infiltrated lymphocytes were preserved in this system for 60 days.^90^ Both methods required customized culture media with varying additives depending on the cell type. The microfluidic method has been applied to establish BTO as a technical part of the 3D bioprinting technique. GBOs generated following Jacob's protocol can be directly co‐cultured with immune cells more conveniently and faster within 1–5 days, providing a platform to develop immunotherapies. This co‐culture method can also be extended to other stromal cells and types of brain tumors.^67^

To efficiently scale up the generation of BTOs and reduce variability within and between batches, a 3D bioprinting technique was applied. This bioprinting technique involves computer‐controlled additive biofabrication, with the potential to build or pattern viable organ‐like structures in 3D using cells and biomaterials. By refining bioinks with key ECM components that propagate cellular viability and bioprinting BTOs in 96‐well plates, BTOs can be used for high‐throughput drug screening after only 7 days of culturing.^125^ In 4D bioprinting, 3D bioprinting is combined with smart materials that respond to stimuli, and this has been used to form 4D organoid arrays. This technique not only allows high‐throughput drug screening, but also reduces manual operation, thus simplifying the process and increasing reproducibility.^82^ However, the ability of these bioprinted organoids to recapitulate cellular heterogeneity and organization comparable to that of parental tumors remains uncertain. In other words, presently, it is more appropriate to regard them as “biofabricated spheroids” until further characterization studies prove their ability to recapitulate their source tissue.^125^, ^126^

## 8 CHALLENGES AND FUTURE PROSPECTS

BTOs are an important new platform for understanding tumor development and developing precision oncology for brain tumors. Below, we detail the current limitations and future prospects of this technology:

1. Accurate recapitulation of brain cellular architecture

Brain tumors mostly occur in adults.^1^ Although the current brain organoids are remarkably similar to the fetal brain, the mature components in brain organoids are insufficient, and neural functions in brain organoids differ from those in the adult brain.^127^, ^128^ Some define “mature” brain organoids as at least 6 months old—a time period in which most NPCs differentiate into neurons and astrocytes and express mature markers.^128^, ^129^ A more extended culture period (>9 months) was proposed to facilitate greater functional maturity, including the formation of dendritic spines and active neuronal networks.^130^ The main reasons for immaturity in BOs include (1) diffusion limitation of culture media, (2) non‐physiological ECM, (3) and missing cell types, such as microglia.^131^ Organoid maturity was positively correlated with BT invasiveness, suggesting that establishing BOs that recapitulate adult brain was critical.^92^ Several optimizations have been made to promote BO maturity, such as the application of human brain ECM to model real ECM,^132^ air‐liquid interface,^133^ microfluidic devices,^132^ and sliced culture to alleviate diffusion.^120^ Moreover, the lack of a functional vascular system is the primary reason for diffusion limitation in BOs. Although the GBOs established by Jacob et al^24^ could maintain some vascular endothelial cells, no fully formed blood vessels were present. Because of this limitation, the use of BOs for testing drugs or CAR‐T cells, which depends on vascular diffusion, is limited. Recently, vasculature in BOs was shown to be created using engineering hESCs to ectopically express hETV2, leading to the acquisition of several blood–brain barrier characteristics and enhanced functional maturation of BOs.^35^ Intracerebral implantation of BOs into immunodeficient mice also generated blood vessels in the BOs.^134^ However, to date, these BOs have not been used in conjunction with BTs. The lack of persistent immune cells is also a defect in BTOs. Co‐culturing could be a solution, as was discussed in this review.

2 Expanding clinical relevance for BOs and BTOs

BTOs could potentially be used to guide personalized therapy in patients with BTs. However, perhaps because of ethical considerations, only two studies have correlated patient drug responses with drug testing in BTOs and responses in patients. Both these studies showed consistency, giving confidence in the wider clinical use of BTOs for patients.^24^, ^78^ Currently, 5 clinical trials on BTOs (NCT04865315, NCT03971812, NCT04868396, NCT03896958, and NCT04478877) are ongoing. BTOs remain a promising tool for precision medicine, but further clinical correlation analysis is required. Thus, more observational research should be performed to reduce ethical risks while expanding the use of this technology.

3 Standardization and automatization of BO and BTO techniques

As emerging state‐of‐the‐art models, techniques for BOs and BTOs are constantly being optimized. No acknowledged standard protocol exists for all BOs or BTOs. Inter‐ and intra‐batch variability are common across studies because BO and BTO generation largely depends on self‐patterning and self‐organization of PSCs/BTSCs without guided differentiation.^21^, ^25^, ^130^, ^135^ The complexity of manual processes in culture is also a major source of variability and error that hampers large‐scale production. Developing standardized protocols and automatized devices will be helpful in ensuring authenticity and expanding the application of precision oncology. Furthermore, organoid factories can be used for high‐throughput drug screening and target investigations. Three‐dimensional bioprinting, computational automatic techniques, and microfluidic techniques can help achieve this goal.

4 Identification of pathogenic factor driving brain tumors

Although the pathogenesis of brain tumors is mainly related to genetic mutations, microenvironmental factors and their relationship with susceptibility are also important factors leading to brain tumors. Ionizing radiation (hazardous factor) and history of allergies (protective factors) are well‐documented risk factors for brain tumors. Other possible risk factors have also been reported by analyzing large clinical databases that require further validation.^136^ Brain organoids can be used to confirm the relationship between tumorigenesis and exposure to risk factors in future studies. For example, exposing brain organoids to hormonal contraception could help determine if associations exist between maternal hormonal contraception use and central nervous system tumors.^137^ Through the genetic manipulation of brain organoids, the relationship between risk factors and genetic susceptibility may also be revealed.

## 9 CONCLUSIONS

Since the BOs and BTOs emerged in 2013 and 2016, the worldwide application of organoid technology has resulted in remarkable advances in the study of precision oncology for brain tumors. In this review, we described the current literature on the establishment of several forms of BTOs and how precisely they modeled different types of brain tumors. Additionally, the promoting effects of BTOs for deeper biological understanding and personalizing therapy for brain tumors are also described. In summary, even though current BTOs are facing some challenges and required optimizations for complete cancer modeling and precision medicine, BTOs are on the way to be indispensable tools for preclinical and clinical research.

## AUTHOR CONTRIBUTIONS

Jie Wen and Xisong liang drafted the manuscript and prepared the figures. Fangkun Liu, Quan Cheng, and Fan Fan collected the related references. Nathaniel Weygant revised the manuscript. Chuntao Li, Liyang Zhang, and Zhixiong Liu generated the organization and designed this review and also revised the manuscript. All authors consented the final manuscripts.

## FUNDING INFORMATION

The Natural Science Foundation of China (NSFC, no. 81402249); the Natural Science Foundation of Hunan Province (no. 2019JJ50963; 2023JJ30972; 2023JJ30897); the Fundamental Research Funds for the Central Universities of Central South University (no. 160171016).

## CONFLICT OF INTEREST STATEMENT

None.

## Data Availability

The data that supports the findings of this study are available in the supplementary material of this article.
